# 2,4-Bis(2-methyl­phen­yl)-3-aza­bicyclo[3.3.1]nonan-9-one *O*-methyl­oxime

**DOI:** 10.1107/S1600536810043436

**Published:** 2010-10-30

**Authors:** P. Parthiban, V. Ramkumar, Yeon Tae Jeong

**Affiliations:** a Department of Image Science and Engineering, Pukyong National University, Busan 608 739, Republic of Korea; b Department of Chemistry, IIT Madras, Chennai, TamilNadu, India

## Abstract

The mol­ecule of the title compound, C_23_H_28_N_2_O, exists in a twin-chair conformation, with equatorial orientation of the *ortho*-tolyl groups on both sides of the secondary amino group. The title oxime compound and its ketone precursor 2,4-bis­(2-methyl­phen­yl)-3-aza­bicyclo­[3.3.1]nonan-9-one exhibit similar stereochemistries, with the orientation of the *o*-tolyl rings almost identical in both compounds. In the title compound, the tolyl rings are at an angle of 23.77 (3)° with respect to one another; the angle in the precursor is 29.4 (1)° [Vijayalakshmi, Parthasarathi, Venkatraj & Jeyaraman (2000 ▶), *Acta Cryst.* C**56**, 1240–1241]. The cyclo­hexane ring and the oxime ether are disordered over two alternative orientations, with a refined site-occupancy ratio of 0.813 (2):0.186 (4). The crystal structure of the title compound is stabilized by inter­molecular N—H⋯π inter­actions.

## Related literature

For the synthesis and biological activities of oxime derivatives of 3-aza­bicyclo­[3.3.1]nonan-9-ones, see: Parthiban *et al.* (2009*a* ▶,*b* ▶, 2010*a* ▶,*b* ▶); Jeyaraman & Avila (1981 ▶). For related structures with similar conformations, see: Vijayalakshmi *et al.* (2000 ▶); Parthiban *et al.* (2009*c* ▶,*d* ▶). For ring-puckering parameters, see: Cremer & Pople (1975 ▶); Nardelli (1983 ▶).
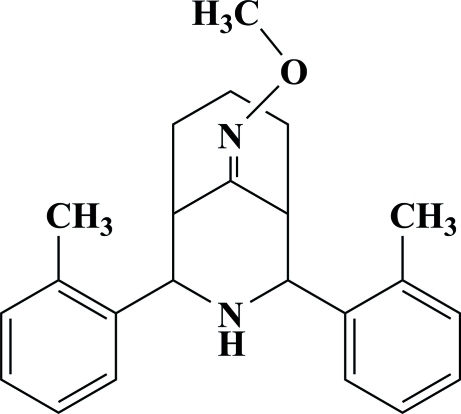


## Experimental

### 

#### Crystal data

C_23_H_28_N_2_O
*M* *_r_* = 348.47
Monoclinic, 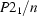

*a* = 6.9700 (9) Å
*b* = 15.3476 (16) Å
*c* = 18.354 (2) Å
β = 94.622 (4)°
*V* = 1957.0 (4) Å^3^
*Z* = 4
Mo *K*α radiation
μ = 0.07 mm^−1^
*T* = 298 K
0.32 × 0.27 × 0.15 mm

#### Data collection

Bruker APEXII CCD area-detector diffractometer
Absorption correction: multi-scan (*SADABS*; Bruker, 2004 ▶) *T*_min_ = 0.977, *T*_max_ = 0.989
12742 measured reflections
4416 independent reflections
2070 reflections with *I* > 2σ(*I*)
*R*_int_ = 0.043

#### Refinement

*R*[*F* ^2^ > 2σ(*F* ^2^)] = 0.060
*wR*(*F* ^2^) = 0.169
*S* = 1.01
4416 reflections
288 parameters
38 restraints
H atoms treated by a mixture of independent and constrained refinement
Δρ_max_ = 0.14 e Å^−3^
Δρ_min_ = −0.19 e Å^−3^

### 

Data collection: *APEX2* (Bruker, 2004 ▶); cell refinement: *APEX2* and *SAINT* (Bruker, 2004 ▶); data reduction: *SAINT* and *XPREP* (Bruker, 2004 ▶); program(s) used to solve structure: *SHELXS97* (Sheldrick, 2008 ▶); program(s) used to refine structure: *SHELXL97* (Sheldrick, 2008 ▶); molecular graphics: *ORTEP-3* (Farrugia, 1997 ▶); software used to prepare material for publication: *SHELXL97*.

## Supplementary Material

Crystal structure: contains datablocks global, I. DOI: 10.1107/S1600536810043436/zl2305sup1.cif

Structure factors: contains datablocks I. DOI: 10.1107/S1600536810043436/zl2305Isup2.hkl

Additional supplementary materials:  crystallographic information; 3D view; checkCIF report

## Figures and Tables

**Fig. 1.**
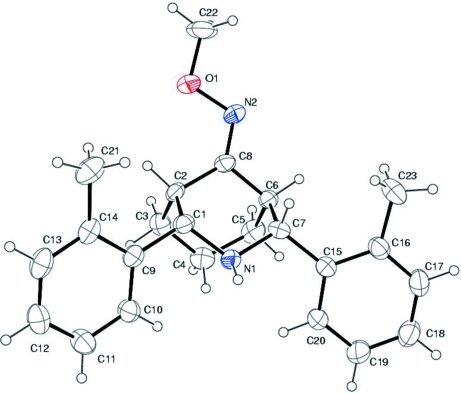
Anistropic displacement representation of the molecule with atoms represented with 30% probability ellipsoids. The minor moiety is omitted for clarity.

**Fig. 2.**
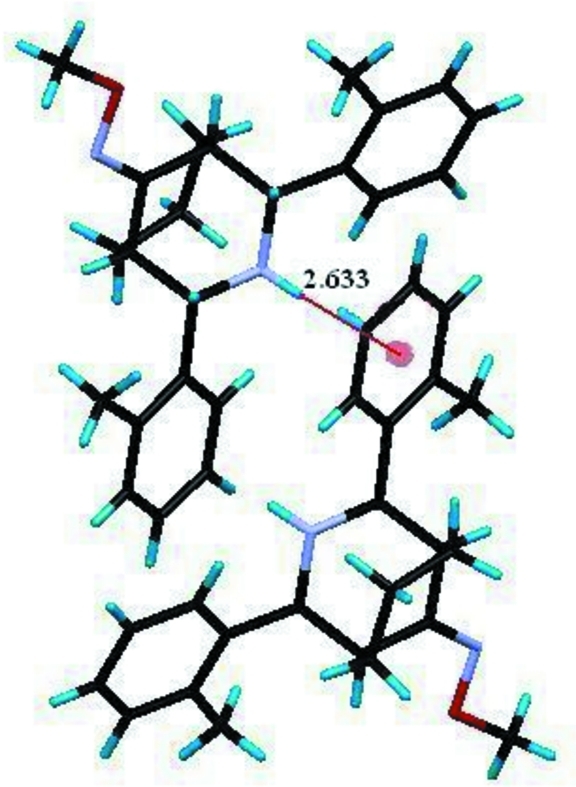
Packing diagram showing the N—H···π interaction. N1—H1···Cg1 = 2.633Å [Cg: C15–C20] and symmetry operator = 1 - x, 2 - y, -z.

**Fig. 3.**
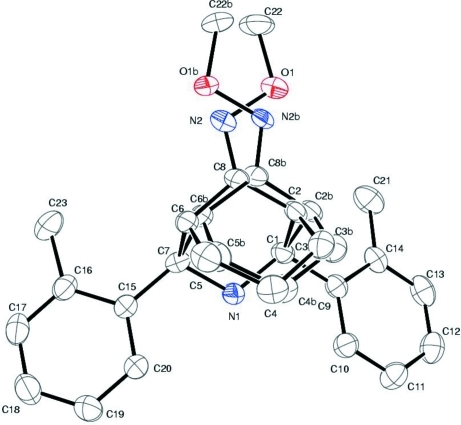
ORTEP (H atoms are removed for clarity) showing the disorder in two orientations.

## supplementary crystallographic information

### Comment

Nitrogen containing heterocyclic oximes and oxime ethers are very important molecules in the field of medicinal chemistry due to their broad spectrum of biological activities *viz.* antifungal, antibacterial, antimycobacterial, analgesic, antagonistic, anticancer, antiinflammatory, local anesthetic and hypotensive activity (Parthiban *et al.*, 2009*a*,*b*, 2010*a*,*b*; Jeyaraman & Avila, 1981). Since the stereochemistry of bio-active molecules is a major criterion for their biological response, it is of immense help to establish the stereochemistry of the newly synthesized molecules. Accordingly, we have synthesized the title oxime ether to examine its conformation and orientation of the substituents.

In the crystal structure, the oxime unit is partially flipped thus inducing disorder for the oxime (N2/O1/C22) and also for the piperidine ring N1/C1/C2/C8/C6/C7 and the cylcohexane ring C2–C6/C8 over two orientations. The site occupancy ratio refined to 0.813 (2) to 0.186 (4). The tolyl rings do not participate in the disorder (Fig. 3).

An analysis of the six-membered piperidine ring gave the following: According to Nardelli (Nardelli, 1983), the smallest displacement asymmetry parameters q_2_ and q_3_ are 0.052 (4) and -0.614 (4) Å, respectively. According to Cremer and Pople (Cremer & Pople, 1975), the ring puckering parameters such as total puckering amplitude Q_T_ and phase angle θ are 0.616 (4) Å and 175.5 (4)°. Thus, all parameters strongly support a near ideal chair conformation for the piperidine ring N1/C1/C2/C8/C6/C7. Similarly, the analysis of cyclohexane ring C2–C6/C8 indicates that it also adopts a chair conformation. It is, however, deviating more from the ideal chair with puckering parameters Q_T_ and θ of 0.553 (7) Å and 169.2 (9)°, and q_2_ and q_3_ of 0.108 (9) and -0.543 (8) Å, respectively.

The torsion angles C8—C6—C7—C15 and C8—C2—C1—C9 of the *ortho*-tolyl rings are -177.2 (3) and 179.2 (3)° and they are orientated at an angle of 23.77 (3)° with respect to one another, whereas in its ketone precursor, 2,4-bis(2-methylphenyl)-3-azabicyclo[3.3.1]nonan-9-one, they are oriented at an angle of 29.4 (1)° (Vijayalakshmi *et al.*, 2000). The crystal structure of the title compound is stabilized by intermolecular N—H···π interactions with N1—H1···*Cg*1 = 2.633 Å, (*Cg*: C15–C20; symmetry operator = 1 - *x*, 2 - *y*, -*z*.)

Thus, the detailed crystallographic study of asymmetry parameters, ring puckering parameters and torsion angles calculated for the title compound proves that the bicyclic moiety exists in a twin-chair conformation with equatorial orientation of the *ortho*-tolyl rings on both sides of the secondary amino group.

### Experimental

The title compound was synthesized by adding 0.501 g *O*-methylhydroxylamine hydrochloride (0.006 mol) and 2.04 g sodium acetate trihydrate (0.015 mol) in a hot ethanolic solution of 1.597 g 2,4-bis(2-methylphenyl)-3-azabicyclo[3.3.1]nonan-9-one (0.005 mol) (Parthiban *et al.*, 2010*b*). The content was refluxed at 345–350 K till completion of the reaction; the progress and completion of the reaction was monitored by TLC. After the consumption of starting material, the content of the flask was concentrated and water was added. Then, the precipitated oxime ether was separated by filtration, washed with an excess of water, and dried in vacuum. X-ray diffraction quality crystals of 2,4-bis(2-methylphenyl)-3-azabicyclo[3.3.1]nonan-9-one *O*-methyloxime were obtained by slow evaporation from ethanol.

### Refinement

The cyclohexane ring C2–C6/C8 and the oxime ether N2/O1/C22 are disordered over two orientations with a refined site occupancy ratio of 0.813 (2) to 0.186 (4). The two moieties were restrained to have similar geometries. The atoms N2b, O1b and C22b of the minor moiety were restrained to have similar anisotropic displacement parameters. The ADPs of all other disordered atoms in the minor moiety were constrained to be identical to those of their counterparts in the major moiety.

The nitrogen H atom was located in a difference Fourier map and refined isotropically. Other H atoms were fixed geometrically and allowed to ride on the parent C atoms with aromatic C—H = 0.93 Å, methylene C—H = 0.97 Å, methine C—H = 0.98 Å and methyl C—H = 0.96 Å. The displacement parameters were set for phenyl, methylene and aliphatic H atoms at *U*_iso_(H) = 1.2*U*_eq_(C) and for methyl H atoms at *U*_iso_(H) = 1.5*U*_eq_(C).

### Crystal data

**Table:**

C_23_H_28_N_2_O	*F*(000) = 752
*M**_r_* = 348.47	*D*_x_ = 1.183 Mg m^−^^3^
Monoclinic, *P*2_1_/*n*	Mo *K*α radiation, λ = 0.71073 Å
Hall symbol: -P 2yn	Cell parameters from 2129 reflections
*a* = 6.9700 (9) Å	θ = 2.2–20.4°
*b* = 15.3476 (16) Å	µ = 0.07 mm^−^^1^
*c* = 18.354 (2) Å	*T* = 298 K
β = 94.622 (4)°	Block, colourless
*V* = 1957.0 (4) Å^3^	0.32 × 0.27 × 0.15 mm
*Z* = 4	

### Data collection

**Table:**

Bruker APEXII CCD area-detector diffractometer	4416 independent reflections
Radiation source: fine-focus sealed tube	2070 reflections with *I* > 2σ(*I*)
graphite	*R*_int_ = 0.043
φ and ω scans	θ_max_ = 28.3°, θ_min_ = 1.7°
Absorption correction: multi-scan (*SADABS*; Bruker, 2004)	*h* = −9→9
*T*_min_ = 0.977, *T*_max_ = 0.989	*k* = −20→13
12742 measured reflections	*l* = −24→20

### Refinement

**Table:**

Refinement on *F*^2^	Primary atom site location: structure-invariant direct methods
Least-squares matrix: full	Secondary atom site location: difference Fourier map
*R*[*F*^2^ > 2σ(*F*^2^)] = 0.060	Hydrogen site location: inferred from neighbouring sites
*wR*(*F*^2^) = 0.169	H atoms treated by a mixture of independent and constrained refinement
*S* = 1.01	*w* = 1/[σ^2^(*F*_o_^2^) + (0.0708*P*)^2^ + 0.1996*P*] where *P* = (*F*_o_^2^ + 2*F*_c_^2^)/3
4416 reflections	(Δ/σ)_max_ < 0.001
288 parameters	Δρ_max_ = 0.14 e Å^−^^3^
38 restraints	Δρ_min_ = −0.19 e Å^−^^3^

### Special details

**Table:**

Geometry. All e.s.d.'s (except the e.s.d. in the dihedral angle between two l.s. planes) are estimated using the full covariance matrix. The cell e.s.d.'s are taken into account individually in the estimation of e.s.d.'s in distances, angles and torsion angles; correlations between e.s.d.'s in cell parameters are only used when they are defined by crystal symmetry. An approximate (isotropic) treatment of cell e.s.d.'s is used for estimating e.s.d.'s involving l.s. planes.
Refinement. Refinement of *F*^2^ against ALL reflections. The weighted *R*-factor *wR* and goodness of fit *S* are based on *F*^2^, conventional *R*-factors *R* are based on *F*, with *F* set to zero for negative *F*^2^. The threshold expression of *F*^2^ > σ(*F*^2^) is used only for calculating *R*-factors(gt) *etc*. and is not relevant to the choice of reflections for refinement. *R*-factors based on *F*^2^ are statistically about twice as large as those based on *F*, and *R*- factors based on ALL data will be even larger.

### Fractional atomic coordinates and isotropic or equivalent isotropic displacement parameters (Å^2^)

**Table:**

	*x*	*y*	*z*	*U*_iso_*/*U*_eq_	Occ. (<1)
N1	0.6085 (3)	0.86925 (11)	0.08349 (9)	0.0470 (5)	
C1	0.5497 (3)	0.81766 (13)	0.14551 (11)	0.0461 (6)	
H1	0.4087	0.8169	0.1428	0.055*	
C7	0.5361 (3)	0.83350 (13)	0.01217 (12)	0.0497 (6)	
H7	0.3953	0.8317	0.0100	0.060*	
C9	0.6212 (3)	0.85822 (13)	0.21810 (12)	0.0462 (6)	
C10	0.7833 (3)	0.91132 (15)	0.22295 (13)	0.0563 (6)	
H10	0.8457	0.9223	0.1810	0.068*	
C11	0.8540 (4)	0.94796 (17)	0.28793 (14)	0.0663 (7)	
H11	0.9614	0.9840	0.2895	0.080*	
C12	0.7663 (4)	0.93131 (18)	0.34982 (15)	0.0704 (8)	
H12	0.8140	0.9552	0.3942	0.084*	
C13	0.6056 (4)	0.87857 (17)	0.34608 (14)	0.0670 (8)	
H13	0.5467	0.8670	0.3887	0.080*	
C14	0.5287 (3)	0.84220 (15)	0.28101 (13)	0.0533 (6)	
C15	0.5949 (3)	0.88982 (14)	−0.05005 (12)	0.0485 (6)	
C16	0.4936 (3)	0.88717 (14)	−0.11942 (13)	0.0554 (6)	
C17	0.5643 (4)	0.93407 (17)	−0.17588 (13)	0.0654 (7)	
H17	0.4987	0.9317	−0.2220	0.078*	
C18	0.7258 (4)	0.98333 (18)	−0.16638 (15)	0.0737 (8)	
H18	0.7717	1.0131	−0.2056	0.088*	
C19	0.8199 (4)	0.9886 (2)	−0.09852 (15)	0.0850 (9)	
H19	0.9277	1.0241	−0.0907	0.102*	
C20	0.7563 (4)	0.94157 (17)	−0.04161 (14)	0.0716 (8)	
H20	0.8242	0.9448	0.0040	0.086*	
C21	0.3492 (4)	0.78778 (18)	0.28148 (15)	0.0799 (9)	
H21A	0.2570	0.8069	0.2431	0.120*	
H21B	0.2956	0.7940	0.3278	0.120*	
H21C	0.3804	0.7277	0.2738	0.120*	
C23	0.3094 (4)	0.8366 (2)	−0.13412 (16)	0.0958 (11)	
H23A	0.3383	0.7757	−0.1376	0.144*	
H23B	0.2439	0.8561	−0.1792	0.144*	
H23C	0.2285	0.8459	−0.0949	0.144*	
C2	0.6198 (9)	0.7228 (3)	0.1364 (2)	0.0452 (10)	0.814 (5)
H2	0.5669	0.6871	0.1743	0.054*	0.814 (5)
C3	0.8388 (9)	0.7096 (8)	0.1418 (3)	0.0547 (14)	0.814 (5)
H3A	0.8937	0.7353	0.1871	0.066*	0.814 (5)
H3B	0.8661	0.6476	0.1439	0.066*	0.814 (5)
C4	0.9363 (12)	0.7491 (12)	0.0785 (4)	0.0608 (14)	0.814 (5)
H4A	1.0673	0.7275	0.0795	0.073*	0.814 (5)
H4B	0.9421	0.8119	0.0844	0.073*	0.814 (5)
C5	0.8307 (10)	0.7274 (7)	0.0047 (3)	0.0628 (12)	0.814 (5)
H5A	0.8582	0.6675	−0.0075	0.075*	0.814 (5)
H5B	0.8804	0.7643	−0.0323	0.075*	0.814 (5)
C6	0.6115 (9)	0.7394 (2)	0.0025 (2)	0.0491 (10)	0.814 (5)
H6	0.5532	0.7158	−0.0438	0.059*	0.814 (5)
C8	0.5378 (8)	0.6901 (3)	0.0635 (2)	0.0475 (11)	0.814 (5)
C2B	0.621 (4)	0.7216 (11)	0.1560 (11)	0.0452 (10)	0.186 (5)
H2B	0.5691	0.6932	0.1980	0.054*	0.186 (5)
C3B	0.843 (4)	0.715 (4)	0.1583 (18)	0.0547 (14)	0.186 (5)
H3C	0.8985	0.7459	0.2011	0.066*	0.186 (5)
H3D	0.8799	0.6543	0.1636	0.066*	0.186 (5)
C4B	0.928 (6)	0.751 (6)	0.091 (2)	0.0608 (14)	0.186 (5)
H4C	1.0571	0.7279	0.0894	0.073*	0.186 (5)
H4D	0.9406	0.8141	0.0970	0.073*	0.186 (5)
C5B	0.814 (5)	0.733 (4)	0.0191 (17)	0.0628 (12)	0.186 (5)
H5C	0.8508	0.6755	0.0023	0.075*	0.186 (5)
H5D	0.8504	0.7749	−0.0167	0.075*	0.186 (5)
C6B	0.594 (5)	0.7349 (11)	0.0214 (12)	0.0491 (10)	0.186 (5)
H6B	0.5385	0.7044	−0.0223	0.059*	0.186 (5)
C8B	0.546 (4)	0.6829 (17)	0.0853 (12)	0.0475 (11)	0.186 (5)
N2	0.4164 (5)	0.6292 (2)	0.0473 (2)	0.0555 (9)	0.814 (5)
O1	0.3550 (4)	0.59007 (17)	0.11187 (14)	0.0658 (9)	0.814 (5)
C22	0.2246 (7)	0.5220 (3)	0.0898 (3)	0.0928 (17)	0.814 (5)
H22A	0.1227	0.5449	0.0570	0.139*	0.814 (5)
H22B	0.1715	0.4978	0.1320	0.139*	0.814 (5)
H22C	0.2916	0.4773	0.0654	0.139*	0.814 (5)
N2B	0.427 (2)	0.6218 (8)	0.0948 (8)	0.048 (4)	0.186 (5)
O1B	0.3413 (16)	0.5918 (7)	0.0264 (5)	0.060 (3)	0.186 (5)
C22B	0.220 (3)	0.5208 (13)	0.0398 (10)	0.087 (7)	0.186 (5)
H22D	0.2958	0.4735	0.0608	0.130*	0.186 (5)
H22E	0.1535	0.5021	−0.0054	0.130*	0.186 (5)
H22F	0.1277	0.5384	0.0731	0.130*	0.186 (5)
H1N	0.563 (4)	0.927 (2)	0.0881 (15)	0.104*	

### Atomic displacement parameters (Å^2^)

**Table:**

	*U*^11^	*U*^22^	*U*^33^	*U*^12^	*U*^13^	*U*^23^
N1	0.0589 (12)	0.0416 (10)	0.0405 (11)	−0.0091 (9)	0.0041 (9)	−0.0006 (9)
C1	0.0432 (13)	0.0455 (12)	0.0500 (14)	−0.0055 (10)	0.0066 (10)	0.0049 (11)
C7	0.0505 (14)	0.0450 (13)	0.0526 (15)	−0.0115 (10)	−0.0024 (11)	−0.0044 (11)
C9	0.0466 (14)	0.0456 (13)	0.0471 (14)	0.0057 (10)	0.0077 (11)	0.0050 (10)
C10	0.0569 (15)	0.0651 (15)	0.0479 (15)	−0.0076 (12)	0.0104 (12)	−0.0052 (12)
C11	0.0605 (17)	0.0771 (18)	0.0612 (18)	−0.0052 (14)	0.0041 (14)	−0.0142 (14)
C12	0.082 (2)	0.0784 (19)	0.0500 (17)	0.0130 (16)	0.0007 (15)	−0.0095 (14)
C13	0.080 (2)	0.0710 (17)	0.0530 (17)	0.0211 (15)	0.0244 (14)	0.0091 (14)
C14	0.0569 (15)	0.0541 (14)	0.0503 (15)	0.0102 (12)	0.0142 (12)	0.0102 (12)
C15	0.0528 (14)	0.0475 (13)	0.0445 (14)	−0.0041 (11)	0.0006 (11)	−0.0035 (11)
C16	0.0612 (16)	0.0500 (14)	0.0529 (15)	0.0001 (12)	−0.0074 (12)	−0.0072 (12)
C17	0.083 (2)	0.0656 (17)	0.0454 (15)	0.0104 (15)	−0.0069 (14)	−0.0010 (13)
C18	0.077 (2)	0.0842 (19)	0.0602 (18)	−0.0015 (16)	0.0084 (16)	0.0196 (15)
C19	0.071 (2)	0.105 (2)	0.077 (2)	−0.0352 (17)	−0.0080 (16)	0.0304 (18)
C20	0.0695 (19)	0.0869 (19)	0.0552 (16)	−0.0322 (15)	−0.0137 (14)	0.0161 (14)
C21	0.074 (2)	0.086 (2)	0.084 (2)	−0.0038 (16)	0.0332 (16)	0.0165 (16)
C23	0.095 (2)	0.102 (2)	0.083 (2)	−0.0286 (19)	−0.0386 (18)	0.0025 (17)
C2	0.0569 (16)	0.0393 (12)	0.040 (3)	−0.0069 (11)	0.011 (2)	0.0081 (16)
C3	0.0604 (17)	0.051 (2)	0.052 (4)	0.0061 (13)	0.002 (2)	−0.005 (3)
C4	0.0500 (17)	0.0626 (19)	0.071 (4)	0.0018 (15)	0.012 (2)	−0.005 (4)
C5	0.075 (2)	0.057 (2)	0.060 (3)	0.0040 (18)	0.025 (2)	−0.008 (3)
C6	0.067 (2)	0.0449 (14)	0.035 (3)	−0.0150 (13)	0.007 (2)	−0.0068 (14)
C8	0.0547 (16)	0.0316 (15)	0.055 (3)	−0.0055 (13)	0.001 (2)	0.004 (2)
C2B	0.0569 (16)	0.0393 (12)	0.040 (3)	−0.0069 (11)	0.011 (2)	0.0081 (16)
C3B	0.0604 (17)	0.051 (2)	0.052 (4)	0.0061 (13)	0.002 (2)	−0.005 (3)
C4B	0.0500 (17)	0.0626 (19)	0.071 (4)	0.0018 (15)	0.012 (2)	−0.005 (4)
C5B	0.075 (2)	0.057 (2)	0.060 (3)	0.0040 (18)	0.025 (2)	−0.008 (3)
C6B	0.067 (2)	0.0449 (14)	0.035 (3)	−0.0150 (13)	0.007 (2)	−0.0068 (14)
C8B	0.0547 (16)	0.0316 (15)	0.055 (3)	−0.0055 (13)	0.001 (2)	0.004 (2)
N2	0.069 (2)	0.0400 (19)	0.057 (2)	−0.0111 (15)	0.003 (2)	0.010 (2)
O1	0.074 (2)	0.0531 (17)	0.0686 (18)	−0.0232 (13)	−0.0030 (13)	0.0137 (12)
C22	0.082 (3)	0.066 (2)	0.126 (4)	−0.041 (2)	−0.020 (3)	0.017 (3)
N2B	0.073 (10)	0.029 (8)	0.042 (8)	−0.011 (6)	0.004 (8)	0.008 (7)
O1B	0.085 (8)	0.045 (6)	0.047 (6)	−0.028 (5)	−0.003 (5)	−0.002 (5)
C22B	0.096 (13)	0.070 (10)	0.090 (14)	−0.050 (9)	−0.018 (13)	0.000 (12)

### Geometric parameters (Å, °)

**Table:**

N1—C7	1.470 (3)	C2—C3	1.536 (5)
N1—C1	1.472 (3)	C2—H2	0.9800
N1—H1N	0.94 (3)	C3—C4	1.519 (5)
C1—C9	1.518 (3)	C3—H3A	0.9700
C1—C2	1.549 (4)	C3—H3B	0.9700
C1—C2B	1.563 (15)	C4—C5	1.525 (5)
C1—H1	0.9800	C4—H4A	0.9700
C7—C15	1.515 (3)	C4—H4B	0.9700
C7—C6	1.552 (4)	C5—C6	1.536 (5)
C7—C6B	1.570 (15)	C5—H5A	0.9700
C7—H7	0.9800	C5—H5B	0.9700
C9—C14	1.389 (3)	C6—C8	1.477 (4)
C9—C10	1.390 (3)	C6—H6	0.9800
C10—C11	1.374 (3)	C8—N2	1.280 (5)
C10—H10	0.9300	C2B—C8B	1.483 (16)
C11—C12	1.357 (4)	C2B—C3B	1.548 (17)
C11—H11	0.9300	C2B—H2B	0.9800
C12—C13	1.380 (4)	C3B—C4B	1.513 (17)
C12—H12	0.9300	C3B—H3C	0.9700
C13—C14	1.386 (3)	C3B—H3D	0.9700
C13—H13	0.9300	C4B—C5B	1.520 (17)
C14—C21	1.505 (3)	C4B—H4C	0.9700
C15—C20	1.375 (3)	C4B—H4D	0.9700
C15—C16	1.406 (3)	C5B—C6B	1.543 (17)
C16—C17	1.385 (3)	C5B—H5C	0.9700
C16—C23	1.506 (3)	C5B—H5D	0.9700
C17—C18	1.355 (4)	C6B—C8B	1.478 (16)
C17—H17	0.9300	C6B—H6B	0.9800
C18—C19	1.362 (3)	C8B—N2B	1.275 (16)
C18—H18	0.9300	N2—O1	1.424 (4)
C19—C20	1.372 (3)	O1—C22	1.422 (4)
C19—H19	0.9300	C22—H22A	0.9600
C20—H20	0.9300	C22—H22B	0.9600
C21—H21A	0.9600	C22—H22C	0.9600
C21—H21B	0.9600	N2B—O1B	1.422 (13)
C21—H21C	0.9600	O1B—C22B	1.414 (14)
C23—H23A	0.9600	C22B—H22D	0.9600
C23—H23B	0.9600	C22B—H22E	0.9600
C23—H23C	0.9600	C22B—H22F	0.9600
C2—C8	1.499 (4)		
			
C7—N1—C1	113.01 (16)	C3—C2—H2	108.0
C7—N1—H1N	109.5 (17)	C1—C2—H2	108.0
C1—N1—H1N	108.5 (18)	C4—C3—C2	113.6 (5)
N1—C1—C9	111.47 (17)	C4—C3—H3A	108.8
N1—C1—C2	108.2 (2)	C2—C3—H3A	108.8
C9—C1—C2	113.3 (2)	C4—C3—H3B	108.8
N1—C1—C2B	119.8 (10)	C2—C3—H3B	108.8
C9—C1—C2B	101.5 (8)	H3A—C3—H3B	107.7
N1—C1—H1	107.9	C3—C4—C5	112.3 (5)
C9—C1—H1	107.9	C3—C4—H4A	109.2
C2—C1—H1	107.9	C5—C4—H4A	109.2
C2B—C1—H1	107.7	C3—C4—H4B	109.2
N1—C7—C15	111.35 (17)	C5—C4—H4B	109.2
N1—C7—C6	110.8 (2)	H4A—C4—H4B	107.9
C15—C7—C6	109.3 (2)	C4—C5—C6	113.9 (5)
N1—C7—C6B	101.4 (9)	C4—C5—H5A	108.8
C15—C7—C6B	123.3 (9)	C6—C5—H5A	108.8
N1—C7—H7	108.5	C4—C5—H5B	108.8
C15—C7—H7	108.5	C6—C5—H5B	108.8
C6—C7—H7	108.5	H5A—C5—H5B	107.7
C6B—C7—H7	102.9	C8—C6—C5	108.9 (4)
C14—C9—C10	118.7 (2)	C8—C6—C7	104.3 (3)
C14—C9—C1	121.0 (2)	C5—C6—C7	117.0 (5)
C10—C9—C1	120.3 (2)	C8—C6—H6	108.8
C11—C10—C9	121.9 (2)	C5—C6—H6	108.8
C11—C10—H10	119.1	C7—C6—H6	108.8
C9—C10—H10	119.1	N2—C8—C6	117.6 (4)
C12—C11—C10	119.7 (3)	N2—C8—C2	130.4 (4)
C12—C11—H11	120.1	C6—C8—C2	112.0 (3)
C10—C11—H11	120.1	C8B—C2B—C3B	106.1 (18)
C11—C12—C13	119.2 (3)	C8B—C2B—C1	100.6 (16)
C11—C12—H12	120.4	C3B—C2B—C1	112 (3)
C13—C12—H12	120.4	C8B—C2B—H2B	112.5
C12—C13—C14	122.4 (2)	C3B—C2B—H2B	112.5
C12—C13—H13	118.8	C1—C2B—H2B	112.5
C14—C13—H13	118.8	C4B—C3B—C2B	114 (2)
C13—C14—C9	118.1 (2)	C4B—C3B—H3C	108.7
C13—C14—C21	118.8 (2)	C2B—C3B—H3C	108.7
C9—C14—C21	123.0 (2)	C4B—C3B—H3D	108.7
C20—C15—C16	117.7 (2)	C2B—C3B—H3D	108.7
C20—C15—C7	120.9 (2)	H3C—C3B—H3D	107.6
C16—C15—C7	121.3 (2)	C3B—C4B—C5B	115 (2)
C17—C16—C15	118.7 (2)	C3B—C4B—H4C	108.5
C17—C16—C23	118.9 (2)	C5B—C4B—H4C	108.5
C15—C16—C23	122.4 (2)	C3B—C4B—H4D	108.5
C18—C17—C16	122.4 (2)	C5B—C4B—H4D	108.5
C18—C17—H17	118.8	H4C—C4B—H4D	107.5
C16—C17—H17	118.8	C4B—C5B—C6B	115 (2)
C17—C18—C19	119.0 (3)	C4B—C5B—H5C	108.5
C17—C18—H18	120.5	C6B—C5B—H5C	108.5
C19—C18—H18	120.5	C4B—C5B—H5D	108.5
C18—C19—C20	120.2 (3)	C6B—C5B—H5D	108.5
C18—C19—H19	119.9	H5C—C5B—H5D	107.5
C20—C19—H19	119.9	C8B—C6B—C5B	107.3 (19)
C19—C20—C15	122.0 (2)	C8B—C6B—C7	122.3 (19)
C19—C20—H20	119.0	C5B—C6B—C7	105 (3)
C15—C20—H20	119.0	C8B—C6B—H6B	107.0
C14—C21—H21A	109.5	C5B—C6B—H6B	107.0
C14—C21—H21B	109.5	C7—C6B—H6B	107.0
H21A—C21—H21B	109.5	N2B—C8B—C6B	134 (2)
C14—C21—H21C	109.5	N2B—C8B—C2B	111.2 (19)
H21A—C21—H21C	109.5	C6B—C8B—C2B	113.1 (16)
H21B—C21—H21C	109.5	C8—N2—O1	110.7 (4)
C16—C23—H23A	109.5	C22—O1—N2	107.5 (3)
C16—C23—H23B	109.5	C8B—N2B—O1B	110.5 (14)
H23A—C23—H23B	109.5	C22B—O1B—N2B	108.2 (11)
C16—C23—H23C	109.5	O1B—C22B—H22D	109.5
H23A—C23—H23C	109.5	O1B—C22B—H22E	109.5
H23B—C23—H23C	109.5	H22D—C22B—H22E	109.5
C8—C2—C3	108.6 (4)	O1B—C22B—H22F	109.5
C8—C2—C1	108.1 (3)	H22D—C22B—H22F	109.5
C3—C2—C1	116.0 (6)	H22E—C22B—H22F	109.5
C8—C2—H2	108.0		
			
C7—N1—C1—C9	−178.06 (17)	C17—C18—C19—C20	2.8 (5)
C7—N1—C1—C2	56.7 (3)	C18—C19—C20—C15	−1.6 (5)
C1—N1—C7—C15	178.36 (18)	C16—C15—C20—C19	−0.9 (4)
C1—N1—C7—C6	−59.8 (3)	C7—C15—C20—C19	175.5 (3)
N1—C1—C9—C14	155.62 (19)	N1—C1—C2—C8	−56.6 (4)
C2—C1—C9—C14	−82.1 (3)	C9—C1—C2—C8	179.3 (3)
N1—C1—C9—C10	−25.4 (3)	N1—C1—C2—C3	65.6 (4)
C2—C1—C9—C10	97.0 (3)	C9—C1—C2—C3	−58.5 (4)
C14—C9—C10—C11	0.1 (3)	C8—C2—C3—C4	53.6 (8)
C1—C9—C10—C11	−178.9 (2)	C1—C2—C3—C4	−68.4 (8)
C9—C10—C11—C12	1.1 (4)	C2—C3—C4—C5	−46.2 (12)
C10—C11—C12—C13	−0.9 (4)	C3—C4—C5—C6	45.7 (13)
C11—C12—C13—C14	−0.5 (4)	C4—C5—C6—C8	−53.0 (9)
C12—C13—C14—C9	1.7 (4)	C4—C5—C6—C7	64.9 (9)
C12—C13—C14—C21	−178.0 (2)	N1—C7—C6—C8	59.8 (4)
C10—C9—C14—C13	−1.4 (3)	C15—C7—C6—C8	−177.2 (3)
C1—C9—C14—C13	177.60 (19)	N1—C7—C6—C5	−60.6 (4)
C10—C9—C14—C21	178.3 (2)	C15—C7—C6—C5	62.4 (4)
C1—C9—C14—C21	−2.7 (3)	C5—C6—C8—N2	−119.0 (6)
N1—C7—C15—C20	25.6 (3)	C7—C6—C8—N2	115.4 (6)
C6—C7—C15—C20	−97.1 (3)	C5—C6—C8—C2	62.0 (6)
N1—C7—C15—C16	−158.2 (2)	C7—C6—C8—C2	−63.6 (5)
C6—C7—C15—C16	79.1 (3)	C3—C2—C8—N2	118.8 (8)
C20—C15—C16—C17	2.1 (3)	C1—C2—C8—N2	−114.6 (6)
C7—C15—C16—C17	−174.2 (2)	C3—C2—C8—C6	−62.4 (6)
C20—C15—C16—C23	−176.7 (3)	C1—C2—C8—C6	64.2 (6)
C7—C15—C16—C23	7.0 (4)	C6—C8—N2—O1	−178.2 (4)
C15—C16—C17—C18	−1.0 (4)	C2—C8—N2—O1	0.6 (8)
C23—C16—C17—C18	177.9 (3)	C8—N2—O1—C22	−178.6 (5)
C16—C17—C18—C19	−1.5 (4)		
